# Refractive Results With EyeCryl Phakic Toric Intraocular Lens Implantation in a Hospital in Medina, Saudi Arabia: A Retrospective Study

**DOI:** 10.7759/cureus.76656

**Published:** 2024-12-31

**Authors:** Ma’an Al-Barry, Esraa K Alshareef, Shatha Albadawi, Aaesha A Alkayyal, Amal N Alharbi, Sumayyah A Alzahrani, Mohammed G Alsaedi

**Affiliations:** 1 Ophthalmology, Taibah University, Medina, SAU; 2 Ophthalmology, Ministry of National Guard, Medina, SAU; 3 Ophthalmology, King Salman Bin Abdulaziz Medical City, Medina, SAU; 4 Ophthalmology, Ohud Hospital, Medina, SAU

**Keywords:** eyecryl, lens, myopia, refractive, toric intraocular lens

## Abstract

Objectives: To evaluate the efficacy, safety, and stability of EyeCryl Phakic intraocular lens (IOL) implantation.

Methodology: This retrospective study was conducted in Maghrabi Hospital in Medina to review 31 patients who underwent posterior chamber phakic IOL (EyeCryl Phakic IOL) for surgical correction of myopia or astigmatism. The data were collected from patient medical records after obtaining their consents.

Results: Our findings demonstrated the efficacy of a toric implantable collamer lens (ICL) for the treatment of myopic astigmatism, with a significant change in all eye measures (sphericity, axis, and cylinder) after IOL implantation for study participants in both right and left eyes. There were no intraoperative complications observed among the studied patients; however, one case required ICL repositioning due to spontaneous axis rotation. The majority of cases showed no postoperative complications except one case complaint of steroid-induced glaucoma. In this study, we observed no significant changes regarding endothelial cell loss.

Conclusion: In summary, EyeCryl Phakic IOL implantation may be safe and effective for myopic astigmatism patients. It improved the visual performance of most of the studied participants without detecting serious complications. Studies with longer follow-ups are needed to observe other complications and ensure the safety of the procedure.

## Introduction

Worldwide, refractive errors are considered the most common cause of visual impairment. Symptoms and signs of refractive errors are the most worrisome and common presentations in general practice in eye clinics. The most common types of refractive error are myopia, hyperopia, astigmatism, and presbyopia. Globally, myopia is considered a public health problem affecting all age groups. Refractive errors can be corrected using eyeglasses, contact lenses, or surgery [1].

There are two established surgical methods for the correction of refractive error: refractive corneal surgery and refractive lens surgery [2]. One of the methods used in refractive surgery is intraocular lens (IOL) implantation, which is generally used in cases that cannot be corrected with corneal surgery [3].

Toric phakic intraocular lens (PIOL) implantation was first proposed by Strambelli in 1954, It is superior to laser refractive surgery for patients with astigmatism and high myopia [4,5]. Their advantages include; high visual quality, less postoperative dry eye, less corneal aberrations, magnification of image, and no risk of corneal ectasia [5].

Eyecryl PIOL is a hydrophilic, foldable, acrylic, plate-haptic, posterior chamber PIOL implant in the sulcus in phakic patients. The lens can be implanted with a 2.75-mm incision and has aspherical optics with a diameter of 4.65 to 5.50 mm in the range of -3.00 to -23.00 diopters [6]. Severe complications may occur after PIOL implantation, such as endothelial decompensation, cataracts, and glaucoma [6].

To the best of our knowledge, there are no published studies in Saudi Arabia describing clinical outcomes following the implantation of this lens. In this study, we retrospectively evaluated the efficacy and safety of EyeCryl posterior chamber PIOL implantation in patients with high myopia and astigmatism.

## Materials and methods

Ethical approval for this study was obtained from the Scientific Research Ethics Committee at Taibah University in Medina (approval number: TU-017-22). A retrospective view was conducted on 31 patients who underwent posterior chamber PIOL (EyeCryl Phakic IOL) implantation for surgical correction of myopia or astigmatism at Maghrabi Hospital in Medina, Western region, Saudi Arabia. Registration as a clinical trial was not required, as the study was a retrospective observation of existing patients’ data. The study adhered to the ethical principles outlined in the Declaration of Helsinki in all stages.

Inclusion criteria were as follows: patients aged between 21 and 40 years who were evaluated with Pentacam topography, myopic astigmatism patients with a spherical equivalent range of -3.00 to -20.00 D and astigmatism -1.00 D at least, corrected distance visual acuity of 20/30 or higher, stable refraction (The change over the previous 12 months is 0.50 D or less), lack of tear film, and ocular surface abnormalities. Furthermore, patients with an anterior chamber angle of 35° or greater, an anterior chamber depth of at least 2.8 mm from the corneal endothelium, and an endothelial cell count of 2500 cells/mm^2^. Patients who do not have corneal scars, ectatic corneal diseases, or retinal diseases. Moreover, patients who had confirmed follow-up visits were included.

We excluded from the study patients younger than 21 years, with a spherical equivalent less than -3.00 and astigmatism -1.00 D, patients with unstable refraction (The change over the previous 12 months is more than 0.50D), and patients with ocular surface abnormalities, corneal, or retinal diseases.

The data were collected, reviewed, and then fed to the Statistical Package for Social Sciences version 21 (SPSS: An IBM Company). Statistical methods used were two-tailed with an alpha level of 0.05 considering significance if the P-value was less than or equal to 0.05. Descriptive analysis was performed by prescribing frequency distribution and percentage for patient variables including demographic data, affected eye, and diagnosis. In addition, post-IOL implantation complications were tabulated. All eye measures before and after IOL implantation were displayed for both right and left eyes as a minimum and, maximum measures with mean and standard deviation. Measures were displayed in graphs. The significance of measure changes was assessed using the non-parametric Wilcoxon test and repeated measures ANOVA.

## Results

A total of 31 participants aged 21-37 years were included, with a mean age of 26.6 ± 4.8 years. Most of the study participants, 24 (77.4%), were females.

The most reported diagnosis was myopic astigmatism 18 (58.1%), followed by myopia 11 (35.5%), while amblyopia and hyperopia were reported in 2 cases. In the vast majority of the cases, 27 (87.1%) had bilateral eye problems. As for lens type, it was Toric EyeCryl among 25 (80.6%) and 6 (19.4%) used implantable collamer lens (ICL) (Table 1).

**Table 1:** Bio-demographic characteristics of study participants undergone IOL implantation ICL: implantable collamer lens; IOL: intraocular lens

Bio-demographic data	N	%
Age in years		
21-25	15	48.4%
26-37	16	51.6%
Gender		
Male	7	22.6%
Female	24	77.4%
Diagnosis		
Myopic astigmatism	18	58.1%
Myopic	11	35.5%
Amblyopia	1	3.2%
Hyperopic both eyes	1	3.2%
Eye		
Right	2	6.5%
Left	2	6.5%
Bilateral	27	87.1%
Lens type		
ICL	6	19.4%
Toric Eyecryl	25	80.6%

There was a significant change in all eye measures (Sphericity, axis, and cylinder) after IOL implantation in both right and left eyes (Table 2).

**Table 2:** Full refraction data (minimum and maximum myopia) before and after IOL implantation P: Wilcoxon test; IOL: intraocular lens *P < 0.05 (significant)

Measures	Before IOL			After IOL			p-value
	Minimum	Maximum	Mean ± SD	Minimum	Maximum	Mean ± SD	
Right eye refraction (Sph)	-3.75	-15.50	-7.10 ± 4.19	-1.00	-2.25	0.08 ± 0.62	0.001*
Left eye refraction (Sph)	-3.75	-13.50	-6.29 ± 4.48	-0.75	-8.00	0-.13 ± 1.60	0.001*
Right eye refraction (CYL)	-3.50	-5.00	-1.76 ± 1.55	-0.25	-1.25	0-.55 ± 0.27	0.005*
Left eye refraction (CYL)	-0.50	-5.00	-2.09 ± 1.28	-0.25	-2.75	0-.80 ± 0.60	0.001*
Right eye refraction (Ax)	1.0	175.0	59.1 ± 59.5	5.0	179.0	74.3 ± 59.2	0.001*
Left eye refraction (Ax)	4.0	180.0	134.9 ± 51.4	2.0	174.0	78.9 ± 64.4	0.001*

**Figure 1:**
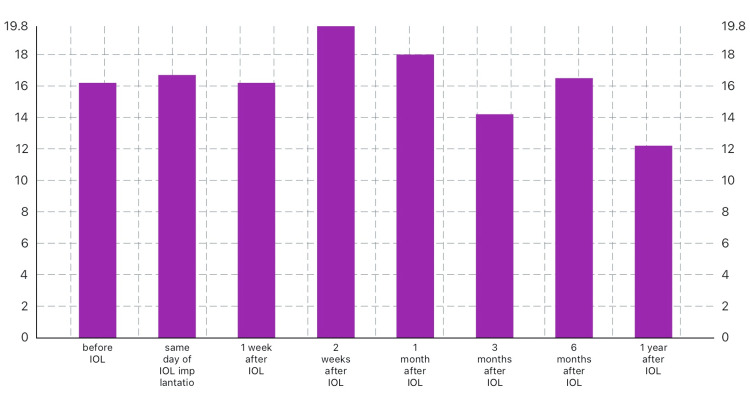
IOP in the right eye before and after IOL implantation IOP: intraocular pressure; IOL: intraocular lens

**Figure 2:**
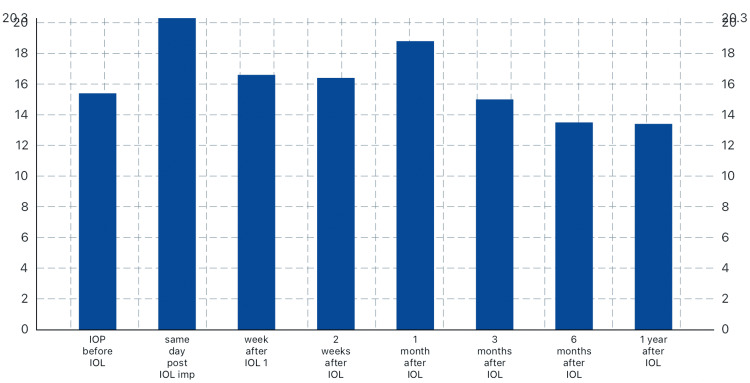
IOP in the left eye before and after IOL implantation IOP: intraocular pressure; IOL: intraocular lens

IOP was significantly decreased in the right eye from 16.2 ± 2.8 before IOL implantation to 14.2 ± 2.8 3 months later and to 12.2 ± 2.5 1 year after implantation with recorded statistical significance (P=0.026) (Table 3). In addition, in Table 4, in the left eye, IOP reduced from 15.4 before IOL to 15.0 after 3 months of IOL implantation and to 13.4 after 1 year (P=0.046) (Figures 1-2).

**Table 3:** Intraocular pressure in the right eye before and after IOL implantation P: Repeated measures ANOVA; IOL: intraocular lens *P < 0.05 (significant)

Phase	Minimum	Maximum	Mean	SD
Before IOL	12.0	24.0	16.2	2.8
Same day as IOL	8.0	33.0	16.7	5.8
1 week after IOL	7.0	27.0	16.2	4.7
2 weeks after IOL	16.0	25.0	19.8	3.0
1 month after IOL	13.0	22.0	18.0	3.7
3 months after IOL	11.0	18.0	14.2	2.8
6 months after IOL	13.0	24.0	16.5	4.5
1 year after IOL	9.0	16.0	12.2	2.5
P-value	0.026*			

**Table 4:** Intraocular pressure in the left eye before and after IOL implantation P: Repeated measures ANOVA; IOL: intraocular lens *P < 0.05 (significant)

Phase	Minimum	Maximum	Mean	SD
Before IOL	10.0	23.0	15.4	3.9
Same day as IOL	6.0	47.0	20.3	9.1
1 week after IOL	10.0	39.0	16.6	5.9
2 weeks after IOL	8.0	26.0	16.4	6.4
1 month after IOL	15.0	22.0	18.8	2.8
3 months after IOL	13.0	17.0	15.0	2.0
6 months after IOL	10.0	22.0	13.5	4.4
1 year after IOL	9.0	19.0	13.4	3.6
P-value	0.046*			

None of the patients’ eyes showed significant changes in their endothelial cell counts after IOL implantation (P > 0.05 for both) (Table 5).

**Table 5:** Endothelial cell counts before and after IOL implantation in right and left eyes P: Wilcoxon test; IOL: intraocular lens *P < 0.05 (significant)

Measures	Before IOL			After IOL			p-value
	Minimum	Maximum	Mean ± SD	Minimum	Maximum	Mean ± SD	
Endothelial cell counts in right eye	2214	3368.0	2726.6 ± 520.9	2375.0	3008.0	2660.4 ± 237.8	0.318
Endothelial cell counts in left eye	2387	3335.0	2736.5 ± 555.4	2238.0	2990.0	2761.2 ± 256.0	0.336

Both eyes showed a significant reduction in corneal thickness after IOL implantation (from 590.2 ± 415.3 to 528.8 ± 47.3 in the right eye (P=0.049), and from 592.0 ± 449.9 to 518.4 ± 58.9 in the left eye (P=0.036)) (Table 6).

**Table 6:** Corneal thickness before and after IOL implantation in right and left eyes P: Wilcoxon test; IOL: intraocular lens *P < 0.05 (significant)

Measures	Before IOL			After IOL			p-value
	Minimum	Maximum	Mean ± SD	Minimum	Maximum	Mean ± SD	
Corneal thickness in right eye	438.0	608.0	590.2 ± 415.3	447.0	588.0	528.8 ± 47.3	0.049*
Corneal thickness in left eye	429.0	616.0	592.0 ± 449.9	420.0	582.0	518.4 ± 58.9	0.036*

Only two patients, one patient had steroid-induced ocular hypertension in both eyes, and one case had toric ICL exchange left eye. One case had a complication immediately after IOL and one had a delayed complication (Table 7).

**Table 7:** Complications among participants who undergone IOL implantation IOL: intraocular lens

Complications	N	%
Complication after surgery		
No	29	93.5
Steroid-induced ocular hypertension in both eyes	1	3.2
Toric implantable collamer lens exchange left eye	1	3.2
Time of complication		
Delayed	1	50.0
Same day	1	50.0

## Discussion

Clinical short and long-term complications that result from lens implantation, such as endothelial cell loss, glaucoma, cataract formation, and pigment dispersion syndrome, are expected to increase with time [7]. In this study, we assessed the refractive results of EyeCryl PIOL implantation by measuring the efficacy, safety, predictability, and stability for the correction of myopic astigmatism, myopia, and two cases with amblyopia and hyperopic OU for one year. There were no intraoperative complications observed among the studied patients; however, one case required ICL repositioning because of spontaneous axis rotation. The majority of cases showed no postoperative complications except one case of steroid-induced ocular hypertension, and no data with axial rotation were detected throughout the 1-year follow-up observation period.

Our findings demonstrated the efficacy of toric ICL for the treatment of myopic astigmatism, with a significant change in all eye measures (sphericity, axis, and cylinder) after IOL implantation in both right and left eyes. Other studies [8-9] have suggested the same findings in assessing the safety and efficacy of ICL in the correction of moderate to severe myopia and even low myopia. In addition, this is in agreement with Choi et al. [9] who reported a more stable visual outcome with toric lens implantation and eliminated the risks of laser treatment. The patient selection process was somewhat different because both Laser-assisted In Situ Keratomileusis (LASIK) and ICL implantation are often performed for low-to-moderate myopia. As a result, we typically discussed the pros and cons of both procedures, as well as their long-term prognosis, visual and refractive results, and cost-effectiveness, before deciding on a specific surgical strategy when both procedures were necessary. With regard to astigmatism degree correction, we observed a statistically significant reduction, especially in the left eyes of the studied patients after IOL implantation, but there was some remaining astigmatism error that remained after surgery. Kamiya et al. [8] revealed this to the spherical ICL, which may have induced small corneal astigmatism. Also, Ichioka et al. [10] detected smaller refractive astigmatism values among the toric group compared with the nontoric group.

In this study, we observed a lower IOP than baseline at 3, 6, and 12 months in the left eye after surgery, whereas in the right eye, the IOP was unstable in the first 6 months and then showed a significant reduction after one year with a P-value of 0.026. Lv et al. [11] observed an early and significant reduction in IOP among mild and moderate myopic patients in the first 7 days postoperatively, and after 90 days in severe myopic patients, they explained this to the anatomical changes in the structure of the eyes. The transient elevation of IOP in the first hours postoperatively may be due to the inflammatory reaction caused by the surgery with the abruption of the blood-aqueous barrier and the remaining lens cortical materials, inflammatory cells, and pigmented debris that resulted in the blockage of the trabecular meshwork [12].

Despite the recent development of posterior IOL, changes in endothelial cell counts are one of the biggest problems among IOL-implanted patients; however, there is a normal annual reduction of EC count within 1% or 2% among those patients, which may be considered to be high [13].In this study, we observed no significant changes in endothelial cell loss in either the right or left eye. This is consistent with many studies that reported no or low endothelial loss in the first year postoperatively with stable endothelial cell density, which indicated that any change in the density may be due to endothelial postoperative remodeling and not related to endothelial damage induced by the lens itself [14]. Hacibekiroglu et al. [15] observed a significant reduction in the EC count in the first two years with a P-value (<0.0001) and no significant loss between the second and fourth years in the long-term observation. Urdem and Agca [3] reported significant EC loss in the first year and no changes in the second-year follow-up. The main cause of this issue is probably mechanical damage to the endothelium due to direct contact with the lens; however, the high rates of patients with EC loss who presented without a history of dislocation or trauma may suggest that other factors influence EC loss among patients with implanted IOL [16].

ICL implantation procedures were performed to correct refractive errors without removing corneal tissue to avoid corneal ectasia. The mean value of corneal thickness decreased gradually until one year postoperatively, with a statistically significant difference preoperatively and postoperatively between the right and left eyes. Dong et al. [17] found a decrease in the densitometry value among high myopic patients compared with normal individuals. They hypothesized this to be due to the increase in endothelial loss among high myopic patients, and this is consistent with our study, we observed a significant decrease in corneal thickness associated with remarkable changes in ECD, whereas Chen et al. [18] detected significant changes in corneal thickness without any changes in ECD.

The study had some limitations, such as the study sample was small. As a result, it may not be representative of the entire population. Furthermore, some patients didn’t adhere to postoperative follow-up and this affected the reliability of data analysis, the assessment of postoperative complications, and vision stability.

## Conclusions

In summary, the EyeCryl PIOL implantation procedure appears to be a safe and effective procedure for patients with myopic astigmatism. The majority of study participants saw an improvement in their visual performance, and no serious side effects were seen. Additionally, none of the patients' eyes showed significant changes in the count of endothelial cells following IOL implantation. Longer follow-up in prospective and clinical trials is essential to better understand the long-term outcomes. Future studies should focus on monitoring complications such as endothelial cell loss and long-term stability and safety. Additionally, exploring the role of inter-ciliary body distance measurement using ultrabiomicroscopy to assess and mitigate the risk of complications.
